# Allergic diseases of the skin and drug allergies – 2015. Randomized controlled, double blind trial of topical twice weekly fluticasone propionate maintenance treatment to reduce risk of relapse in mild or moderate atopic dermatitis in children

**DOI:** 10.1186/1939-4551-6-S1-P102

**Published:** 2013-04-23

**Authors:** Elena Rubio, Inocencia Martinez-Mir, Francisco J Morales-Olivas, Antonio Martorell-Aragonés, Vicente Palop Larrea, Alejandro Bernalte Sesé, Juan C Cerdá Mir, Pedro Polo Martín, Isabel Febrer, Laura Aranda Grau, Inmaculada Llosa Cortes, María José Tejedor Sanz, Juan C Juliá Benito, Teresa Álvarez De Laviada, María Victoria Planelles Cantarino, Esther Apolinar Valiente, Mercedes Loriente Tur, Antonio M Abella Bazataqui, Irene Álvarez Gonzalez, Cristina Morales-Carpi, Maria Enrriqueta Burches Greu, Ana B Ferrer Bautista, Raúl Felix Toledo, Dolores Marmeneu Laguia, Victoria E Garcia-Martinez, María Antonia Beltran Marques, María Begoña Rodriguez Gracia, Inocencia Martinez-Mir

**Affiliations:** 1 Clinical Pharmacology Unit, Hospital, Valencia, Spain; 2 Dirección Médica-Fundación Hgu, Chguv, Valencia, Valencia, Spain; 3 Departamento Farmacología, Universitat De Valéncia, Valencia, Spain; 4 Unidad De Alergía Pediátrica, Chguv, Valencia, Spain; 5 Subdirección Médica Asistencial, Departamento De Salud La Ribera, Valencia, Spain; 6 Servicio De Farmacia, Chguv, Valencia, Spain; 7 Centro De Salud (CS) Barrio De La Luz, Xirivella, Valencia, Xirivella, Spain; 8 Servicio Dermatología, Chguv, Valencia, Spain; 9 Centro De Atención Primaria De Pobla De Vallbona. Valencia, Spain; 10 Monserrat, Valencia, Monserrat, Spain; 11 CS Torrente II, Valencia, Torrent, Spain; 12 CS Alzira, Valencia, Alzira, Spain; 13 Centro De Salud (CS) Barrio De La Luz, Xirivella, Valencia, Xirivella, Spain; 14 Paiporta, Valencia, Paiporta, Spain; 15 CS Torrente I, Valencia, Torrent, Spain; 16 CS Alacuas, Valencia, Alacuas, Spain; 17 Centro De Atención Primaria De Pobla De Vallbona, Valencia, Pobla de Vallbona, Spain; 18 CS Burjasot II, Valencia, Burjasot, Spain; 19 Picasent, Valencia, Picasent, Spain; 20 CS Xirivella, Valencia, Xirivella, Spain; 21 CS Auxiliar El Vedat De Torrent, Valencia, Torrent, Spain

## Background

One of the most troublesome features of atopic dermatitis (AD) is its chronic relapsing nature. The long-term efficacy and potential of corticosteroids to reduce or prevent relapses have only partially been addressed, especially in children. This study was designed to compare an intermittent dosing regimen of fluticasone propionate (FP) cream 0.05% (twice per week) with its vehicle base in reducing the risk of relapse in paediatric subjects with stabilized AD.

## Methods

Randomized controlled, multicentric, double blind trial was conducted**.** Severity of AD was scored by means of the modified Scoring of Atopic Dermatitis system (SCORAD). Children (2-10 years) with mild or moderate AD (SCORAD up to 50; exclusion criteria were >30% affected body surface area and/or head) were enrolled into an Open-label Stabilization Phase (OSP) of up to 2 weeks on twice daily FP. Those children who achieved treatment success (SCORAD<5or≥75% initial SCORAD) entered the Double-blind Maintenance Phase (DMP). They were randomly allocated to receive FP or vehicle twice weekly on consecutive days for 16 weeks. The primary study endpoint was relapse rate of AD, time to relapse and severity of disease were also studied. Kaplan–Meier estimates were calculated to describe the time to relapse distribution. The study protocol was approved by the Ethics Committee and all patients’ parents provided their written informed consent.

## Results

Fifty-four patients (29 girls) entered the OSP (23 mild AD) and 49 (26 girls) continued into the DMP. Mean age was 5.5 (SD2.8) and 5.1 (SD2.3) yrs for FP and vehicle groups, respectively. Four patients withdrew from the DMP (two in the FP group and two in the vehicle group). Patients treated with FP twice weekly had a 2.7 fold lower risk of experiencing a relapse than patients treated with vehicle (relative risk 2.72, SE1.28; p=0.034). FP was also superior to vehicle for delaying time to relapse (Mean 108.4 SD32.5 and Mean 77.4 SD54.6, respectively). Therapy with both treatments was well tolerated.

## Conclusions

This long-term study shows that twice per week FP provides an effective maintenance treatment regimen to control AD through a significantly reduction of the risk of relapse in children with mild and moderate AD.

Grant ISCIII EC08/00004.

